# Network analysis identifies core fear symptoms as screening clues for post-PCI rehabilitation in patients with acute myocardial infarction

**DOI:** 10.3389/fcvm.2026.1805006

**Published:** 2026-06-15

**Authors:** YanRong Gu, XiaoMin Wu, YiYi Chai, Ping Lin, XueQin Gao, YiNi Wang, GuoJie Liu, ZhenJuan Zhao, Ling Li

**Affiliations:** 1 Department of Cardiology, Second Affiliated Hospital of Harbin Medical University Harbin, Harbin, China; 2 Department of Cardiology, Harbin Medical University Harbin, Harbin, China; 3 Ningbo Yinzhou Qianhu Hospital, Ningbo, Zhejiang, China

**Keywords:** acute myocardial infarction, fear of disease progression, kinesiophobia, network analysis, pain catastrophizing, percutaneous coronary intervention

## Abstract

**Background:**

Acute myocardial infarction is a leading cause of death among cardiovascular patients. Fear following percutaneous coronary intervention (PCI) can impede patient recovery. Thus, identifying the core and key symptoms of patients' fear and formulating corresponding countermeasures are essential.

**Objective:**

To analyze the key fear symptoms in acute myocardial infarction patients post—PCI and provide evidence for precision risk screening and supportive care for post-PCI AMI patients.

**Methods:**

A cross—sectional survey was conducted. Using convenient sampling, patients who underwent PCI for acute myocardial infarction at a Harbin tertiary hospital's cardiology department from June to October 2024 were enrolled. Data were collected via the Pain Catastrophizing Scale, Tampa Scale for Kinesiophobia Heart, and Fear of Progression Questionnaire-Short Form. Network analysis, including centrality calculations and stability estimations, was performed using R software.

**Results:**

The strongest connection was found between “Anxiety causes physical symptoms such as rapid heart rate, stomach pain, and nervousness” and “Relying on strangers for daily life makes me anxious,” serving as the most crucial bridging symptom. “Exercise aggravates heart problems” had the highest expected influence (EI = 1.279), was centrally located in the network, and was identified as the core fear symptom in post—PCI acute myocardial infarction patients.

**Conclusion:**

This study employed network analysis to identify closely—linked and highly predictive symptoms within the post—PCI fear network of acute myocardial infarction patients. The core symptom “Exercise aggravates heart problems” may serve as an exploratory screening clue, and healthcare workers could use this to develop stratified screening and personalized supportive plans to improve patient prognosis.

## 1 Introduction

Acute myocardial infarction (AMI) is a condition in which the heart muscle suffers injury due to ischemia and hypoxia. It remains one of the primary causes of death among cardiovascular patients (1, 2). In 2020, out of the patients hospitalized for coronary heart disease in China, 789,215 individuals (accounting for 14.9%) were diagnosed with myocardial infarction. The mortality rate was 4.3%, the average duration of hospitalization was 8.5 days, and the average total cost amounted to 35,614.4 RMB (3). According to the report released by the American Heart Association (AHA) in 2024, the annual number of deaths caused by AMI in the United States is approximately 1.5 million, and about 450,000 deaths are attributed to recurrent myocardial infarction (4). Percutaneous coronary intervention (PCI) for the purpose of revascularization has emerged as a crucial measure for saving the lives of patients with coronary artery disease (5). Nevertheless, even after undergoing PCI, there persists a risk of adverse cardiac events (6). AMI patients are highly vulnerable to experiencing negative psychological states, including pessimism, disappointment, anxiety, depression, and fear, as a result of the stress imposed by the disease (7). Such negative psychological conditions can impede the recovery process of AMI patients and intensify the physical symptoms of the disease that they perceive (8). Specifically, the presence of fear can dampen patients' motivation for exercise rehabilitation, which may subsequently lead to muscle atrophy, loss of muscle strength, and an elevated risk of recurrent cardiovascular diseases. Therefore, addressing the fear experienced by AMI patients is of equal importance to the management of their physical symptoms (9).

The “fear-avoidance” model (10) posits that individuals exposed to a painful stimulus tend to display two distinct behavioral responses: “confrontation” and “avoidance.” Those who perceive painful stimuli in a catastrophic manner and employ a series of “avoidance” strategies are likely to develop kinesiophobia (11). Chronic kinesiophobia can impede the recovery process, thereby intensifying the fear of disease progression (12). Kinesiophobia is defined as the fear of causing harm or re-injury to the body during physical activities or exercises. It represents an excessive and irrational apprehension towards physical activity or exercise, stemming from the concern of inflicting injury or re-injury on the body (13). The concept of Fear of Progression was first introduced by Dankert (14). It refers to the patient's anxiety regarding the various adverse outcomes associated with the further advancement and recurrence of the disease, encompassing biological, psychological, and social dimensions. Pain catastrophizing, on the other hand, is a psychological state in which patients are preoccupied with actual or anticipated pain, exhibit an exaggerated reaction to it, or feel a sense of helplessness in coping with it (15).

Acute myocardial infarction (AMI) patients often exhibit various fear symptoms. Previous studies on fear in AMI patients mainly concentrated on the current situation and analysis of influencing factors (16), yet they overlooked the interconnections and functions of specific symptoms within different fear manifestations. Network analysis (17) is grounded in the interactions among the basic components of psychological phenomena. It models patterns within psychological aspects, including symptoms, traits, emotions, behaviors, cognitions, desires, abilities, and environmental factors. Through network nodes and edges, it analyzes the relationships between different dimensions (18). This method helps identify both core and bridging symptoms (19). Identification of core and bridging symptoms may help prioritize screening and supportive strategies for the whole symptom network. Compared with traditional total score analysis of psychological scales, network analysis can visualize the interactive connections and hierarchical structure among individual fear symptoms, rather than merely summarizing an overall score. This method is especially suitable for post-PCI AMI patients who present with complex, co-occurring psychological and physical symptoms, helping to identify core and bridge symptoms that drive the whole fear network, thus supporting precise and targeted clinical interventions. Therefore, based on the “fear—avoidance” model (10), this study employed network analysis and visualized the network using the R language. The aim was to identify the core and key fear symptoms in post—percutaneous coronary intervention (PCI) AMI patients and propose countermeasures, thereby providing a basis for precise clinical intervention and care.

## 2 Methods

### 2.1 Design and participants

A cross-sectional survey was conducted, and patients with acute myocardial infarction who had undergone percutaneous coronary intervention (PCI) in the Department of Cardiovascular Medicine at the Second Affiliated Hospital of Harbin Medical University were conveniently sampled for this study from June 2024 to October 2024.

Inclusion criteria: (Ⅰ) Aged 18 years or older; (Ⅱ) Meeting the diagnostic criteria for acute myocardial infarction (AMI) specified in the Diagnostic and Therapeutic Guidelines for Acute ST-Segment Elevation Myocardial Infarction; (Ⅲ) Having normal physical activity post-PCI; (Ⅳ) Being conscious with normal reading and writing abilities; (Ⅴ) Providing informed consent and voluntarily participating in the study.

Exclusion criteria: (Ⅰ) Individuals with respiratory failure, chronic renal failure, tumors, or other serious comorbidities; (Ⅱ) Those who had participated in a similar study within the past 6 months or were currently participating in one; (Ⅲ) Participants who had recently experienced other negative life events, such as the loss of a loved one, accidental injuries, natural disasters, etc.

The study was carried out after obtaining informed consent from the patients, and they signed the informed consent forms. The questionnaire survey was conducted on a one-to-one basis. Given that some of the study participants had limited literacy skills, the researcher asked the questions and filled out the questionnaires uniformly according to the participants' answers. The researcher was well-versed in the application of the survey tools and provided standardized and consistent instructions. Objective indicators and closed-ended questions were utilized as extensively as possible. The investigator was present during the entire process, collecting and reviewing the questionnaires on the spot.

### 2.2 Measures

#### 2.2.1 Demographic information

This included socio—demographic information, namely age, gender, education level, marital status, occupational status, per capita monthly household income, family location, mode of residence, and the main medical payment method. Additionally, it covered aspects such as smoking history, Body Mass Index (BMI), the number of coronary stent implantations, the occurrence of acute cardiac events, and the presence of comorbid chronic diseases.

#### 2.2.2 Pain catastrophizing scale (PCS)

The Chinese version of the scale, which was standardized by Yap et al. (20) in 2008, is a 13-item scale that measures the three dimensions of rumination (items 8, 9, 10, and 11), exaggeration (items 6, 7, and 13), and helplessness (items 1, 2, 3, 4, 5, and 12), in which patients rate the degree to which they think and feel about their experiences of actual or perceived pain using the Likert5 scale. The scale was scored using the Likert5 scale, ranging from 0 for “never” to 4 for “always”, with total scores ranging from 0 to 52, the higher the score, the higher the level of pain catastrophizing, and the scale had good reliability and validity. The reliability of this scale is good, the total scale Cronbach's alpha is 0.927, and the dimensions Cronbach's alpha is 0.809, 0.768, 0.839 respectively.

#### 2.2.3 Tampa scale for kinesiophobia heart (TSK-SV heart)

The Tampa Scale for Kinesiophobia Heart (TSK-SV Heart) was adapted by Swedish scholars Bäck et al. (21) in 2013 based on the Chronic Pain Patient's Exercise Fear Scale. In 2019, Chinese scholars (22) Chineseized it and used it to measure the level of cardiac disease patients' fear of exercise, and the total Cronbach's *α* coefficient of the scale was 0.859, and the content validity index was 0.824, with good reliability. The scale consisted of 4 dimensions, including risk perception (items 3, 8, 11, 16), exercise fear (items 1, 7, 9, 13), exercise avoidance (items 2, 4, 12, 14, 17), and dysfunction (items 5, 6, 10, 15), with a total of 17 items. The scale was scored on a 4-point Likert scale from 1 for “strongly disagree” to 4 for “strongly agree”, with entries 4, 8, 12, and 16 being reverse scored, and the total score ranged from 17 to 68, with the higher the score, the higher the level of fear of exercise. The higher the score, the higher the level of exercise fear. The higher the score, the higher the level of fear of sports. If the total score is >37, the patient can be evaluated as a patient with fear of sports. The total Cronbach's alpha coefficient of the questionnaire in this study was 0.774.

#### 2.2.4 Fear of progression questionnaire-short form (FoP-Q-SF)

This scale was compiled by Mehnert et al. (23) on the basis of the FoP-Q, still using the Likert 5-point scale, with the entries reduced to 12, and a total score of 12–60, with higher scores indicating a greater degree of fear of disease progression. In the present study, the Chinese version of FoP-Q-SF was used to measure the fear of disease progression in AMI patients (24). The scale is a self-assessment scale, which contains two latitudes: social family and physical health, with a total of 12 entries and a total score of 12–60 points, and the higher the total score, the more serious the fear of disease progression is, and there is a dysfunction of the fear of disease progression. A total score of ≥34 indicates that the fear of disease progression of AMI patients exceeds the normal level and there is a dysfunction of the fear of disease progression psychology, and the Cronbach's *α* of this scale is 0.886, which has good internal consistency. In this study, we conducted item-level network analysis instead of dimension-level analysis, because network analysis focuses on interactions between individual symptoms rather than composite dimension scores. Item-level analysis can reveal more precise and clinically meaningful connections among specific fear symptoms in post-PCI AMI patients.

### 2.3 Statistical procedures

All data were analyzed using the Statistical Package for the Social Sciences (SPSS 27.0) and R statistical software (version 4.4.3). Descriptive analysis was performed to report the sample characteristics. For continuous variables, means and standard deviations (SD) were calculated; for categorical variables, frequencies and percentages were determined.

Network analysis of fear symptoms in patients after PCI for acute myocardial infarction was performed using the qgraph package in R 4.4.3 software. Symptom network graphs were constructed based on the EBICglasso function (25). The network was regularized using the Least Absolute Shrinkage with Selection Operator (LASSO). False positives were removed by reducing the marginal coefficients of the weaker or spurious estimates to zero. Extended Bayesian Information Criterion (EBIC) is combined to select the optimal model. The dimensional network consists of nodes and edges, with thicker and darker edges indicating stronger correlation between dimensions.

The influence of each node was analyzed by calculating the node's Expected Influence (EI). The EI represents the sum of the weighted values of the nodes that are directly connected to other nodes. It is the most commonly employed centrality metric, and it is more stable than other traditional centrality statistics such as strength, closeness, and proximity (26). A higher expected influence value implies that the node exerts a greater influence on its directly adjacent nodes and holds a crucial position within the network.

The Centrality Stability Coefficient (CS-coefficient) was estimated. A CS-coefficient greater than 0.5 indicates that the network model has good stability, while a value greater than 0.25 suggests that the stability of the network model is acceptable (27). The Bootstrap algorithm was employed to estimate the 95% confidence intervals, aiming to assess the accuracy of the edge weights and centrality indicators. Statistical significance was considered when the *P*-value was less than 0.05. Although the CS coefficient indicated acceptable stability, the present network included more than 40 nodes, and the sample size of 449 may be relatively limited for estimating such a high-dimensional network. Results should be interpreted with caution.

To formally assess potential item and construct redundancy across the three fear-related scales, we performed both inter-item correlation analysis and reliability-based redundancy checks. The Pearson correlation coefficients between all items ranged from 0.100 to 0.360, with no pairs exceeding the commonly accepted threshold of *r* ≥ 0.70 for high redundancy. Additionally, the corrected item-total correlations (CITC) ranged from 0.188 to 0.370, and no substantial increase in Cronbach's *α* was observed when any single construct was removed. Collectively, these findings indicate no severe item or construct overlap, supporting the retention of all items for the item-level network analysis.

## 3 Results

### 3.1 Descriptive statistics

A total of 498 questionnaires were distributed, and 449 questionnaires were eventually recovered effectively, resulting in an effective recovery rate of 90.16%. Elderly patients made up a relatively large proportion. Specifically, 244 patients (54.3%) were 60 years old or above, 165 patients (36.7%) were aged between 45 and 59 years old, and 40 patients (8.9%) were 44 years old or younger. Among these respondents, 307 (68.4%) were male and 142 (31.6%) were female. Other general information is presented in Table 1.

**Table 1:** Demographic and clinical information of the sample (*n* = 449).

Variable	Classification	*n*	%
Age	≤44	40	8.9%
	45–59	165	36.7%
	≥60	244	54.3%
Gender	Male	307	68.4%
	Female	142	31.6%
Body mass index (BMI)	<18.5	13	2.9%
	18.5 ≤ BMI < 24.0	136	30.3%
	24.0 ≤ BMI < 28.0	195	43.4%
	BMI > 28.0	105	23.4%
Smoking	Never	167	37.2%
	Quit	112	24.9%
	Smoking	170	37.9%
Drinking	No	236	52.6%
	Occasionally	122	27.2%
	Drink every day	68	15.1%
	Quit drinking	23	5.1%
Constipation	No	323	71.9%
	Yes	126	28.1%
Place of residence	Urban	260	57.9%
	Rural	189	42.1%
Marital status	Unmarried	6	1.3%
	Married	404	90.0%
	Divorced	11	2.4%
	Widowed	28	6.2%
Child	0	17	3.8%
	1	231	51.4%
	2	155	34.5%
	≥3	46	10.2%
Education level	Elementary school and below	136	30.3%
	Junior high school	167	37.2%
	High school or junior college	88	19.6%
	College or above	58	12.9%
Monthly household income per capita (RMB)	≤3,000	224	49.9%
	3,000–5,000	161	35.9%
	≥5,000	64	14.3%
Chronic comorbidity	No	114	25.4%
	Yes	335	74.6%
Disease duration (years)	<1	329	73.3%
	1–5	84	18.7%
	6–10	26	5.8%
	>10	10	2.2%

### 3.2 Network structure

**Figure 1:**
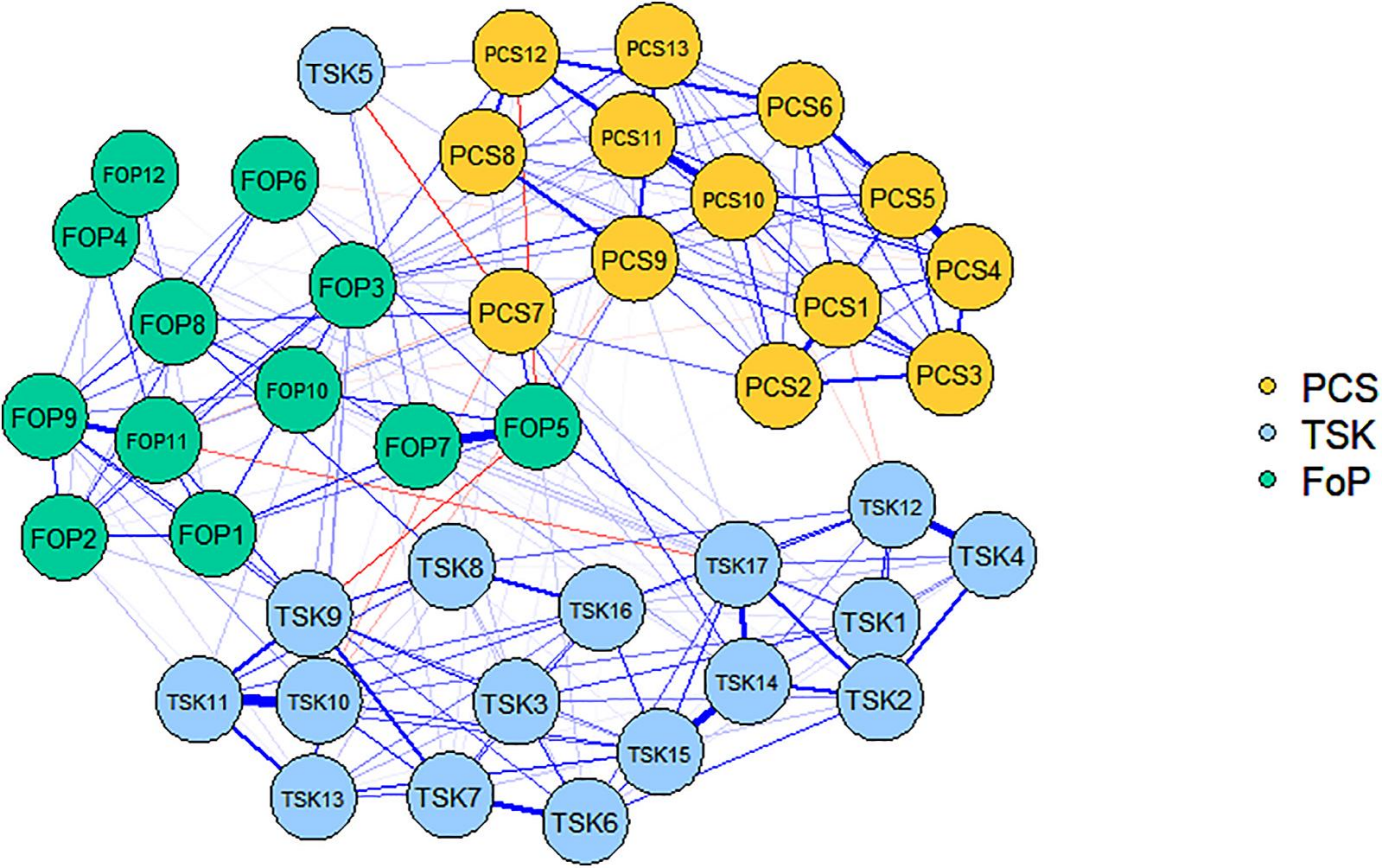
Networks of pain catastrophizing, kinesiophobia, and fear of progression in patients with acute myocardial infarction. The thickness of an edge indicates the degree of correlation between two nodes. Blue edges represent positive correlations, and red edges represent negative correlations.

As depicted in Figure 1, the network contains a total of 237 non-zero interconnected edges. Table 2 presents the Expected Influence (EI), Bridge Expected Influence (BEI), and predictability of the nodes within the network. The node with the highest EI is TSK2: “Exercise aggravates heart problems” (EI = 1.279), followed by PCS10: “Can't help thinking this is going to hurt” (EI = 1.180) and B9: “Fear that I might accidentally injure myself” (EI = 1.180). Another notable node is “Accidentally injure myself” with an EI of 1.081 (Table 2, Supplementary Figure S1).

**Table 2:** Expected influence, bridge expected influence and predictability of all the nodes included in mixed graphical model.

Items	Expected influence	Bridge expected influence	Predictability
PCS1: I'm always worrying if the pain will stop.	0.985	−0.034	0.773
PCS2: I don't think I'm gonna make it.	0.986	0.061	0.739
PCS3: I fell too hard and think it would never get better	0.953	0.000	0.728
PCS4: I feel that the pain is stronger than I am and that's terrible	1.040	0.006	0.807
PCS5: I don't think I can take this pain anymore.	1.073	0.044	0.799
PCS6: I'm afraid the pain will get worse.	0.955	0.030	0.692
PCS7: I can't stop thinking about other painful experiences.	0.469	0.270	0.347
PCS8: I waited anxiously for the pain to go away.	0.743	0.000	0.544
PCS9: I can't distract myself from the pain.	1.039	0.041	0.694
PCS10: I couldn't help but think: this is going to hurt.	1.180	0.065	0.804
PCS11: I can't help but think: let the pain go away once and for all.	1.027	0.088	0.748
PCS12: There's nothing I can do to ease the pain.	0.674	0.021	0.558
PCS13: Suspecting that something is going to go terribly wrong with me.	0.722	0.091	0.520
TSK1: I'm afraid of hurting myself during physical activity/exercise.	0.854	0.054	0.603
TSK2: If I try to be physically active/exercise, the heart problem worsens	1.279	0.012	0.744
TSK3: My body suggests that something is seriously wrong with me.	0.718	0.009	0.394
TSK4: Heart problems may subside if I am physically active/exercise.	0.749	0.000	0.539
TSK5: People (family/friends, etc.) don't take my health seriously enough.	0.033	0.010	0.135
TSK6: My heart problems will gradually weaken me.	0.837	0.096	0.506
TSK7: Heart problems are often due to damage to the body.	0.847	0.037	0.472
TSK8: Just because something causes discomfort in the chest doesn't mean it's dangerous	0.635	0.106	0.298
TSK9: I'm afraid I'll hurt myself by accident.	1.081	0.176	0.595
TSK10: By avoiding unnecessary exercise, I can prevent heart problems from getting worse	1.068	0.031	0.649
TSK11: I wouldn't have heart problems if nothing dangerous was happening in my body	1.045	0.049	0.617
TSK12: I can manage heart problems better if I am physically active/exercising	0.858	−0.038	0.610
TSK13: Heart problems suggest when I should stop physical activity/exercise so I don't hurt myself	0.678	0.048	0.409
TSK14: Physical activity/exercise really isn't safe for someone in my physical condition	1.080	0.172	0.677
TSK15: I'm too prone to heart problems to do the same with others	1.053	0.000	0.667
TSK16: Although some of these things have caused me a lot of heart problems, I don't think they're really dangerous	0.836	0.000	0.418
TSK17: People with heart problems I should avoid exercising	1.078	0.138	0.682
FoP1: I become anxious at the thought that the disease may progress	0.763	0.073	0.312
FoP2: I am nervous before doctor's appointments or regular checkups	0.640	0.069	0.324
FoP3: I am afraid of the pain caused by this disease	0.916	0.535	0.464
FoP4: I worry that I am less productive because of the disease	0.997	0.015	0.823
FoP5: I get some physical discomfort when I am anxious	0.883	0.179	0.487
FoP6: I worry about passing the disease on to my children	0.369	0.020	0.165
FoP7: The need to rely on strangers for daily living makes me anxious	0.729	0.132	0.420
FoP8: Worrying about not being able to continue my hobbies because of my illness	0.740	0.210	0.315
FoP9: Worrying about major treatments in the course of my illness	1.018	0.143	0.415
FoP10: Worrying about medication damaging my body	0.426	0.078	0.269
FoP11: Worrying about what will happen to my family if I have a problem	0.752	0.075	0.393
FoP12: The idea of not being able to work because of my illness bothers me	0.919	0	0.815

Regarding predictability, the highest value was observed in FoP4: “I worry that I am less productive because of the disease” (predictability = 0.823), succeeded by FoP12: “The idea of not being able to work because of my illness bothers me” (predictability = 0.815) and PCS4: “Feeling that the pain is stronger than me” (predictability = 0.807).

Among the nodes, those with the highest Bridge Expected Influence (BEI) were FoP3: “I am afraid of the pain caused by this disease” (BEI = 0.535), PCS7: “Constantly thinking back to another painful experience” (BEI = 0.270), and FoP8: “Worrying about not being able to continue my hobbies because of my illness” (BEI = 0.210).

### 3.3 Network stability and accuracy

Regarding the assessment of network stability and accuracy, the results indicated that the CS value of the network's expected influence was 0.75 (>0.5) (Supplementary Figure S2), suggesting that the network had good stability. Additionally, the CS value of the bridges' expected influence was 0.67 (>0.5) (Supplementary Figure S2), which demonstrated that the network was stable enough.

The variability of the edge weights was investigated by estimating the 95% confidence intervals through the Bootstrap method. The outcomes of 1,000 resamplings from the sample pool (Supplementary Figure S3) revealed that the 95% confidence intervals obtained by Bootstrap were narrow, indicating a high level of accuracy for both the edge weights and the centrality metrics. Supplementary Figure S4 illustrates the Bootstrap variance test for the expected influence of the nodes. Given the relatively high dimensionality (>40 nodes) of the network compared to the sample size, high expected influence reflects strong connectivity within the cross-sectional network rather than causal importance or predictive validity.

## 4 Discussion

To our knowledge, the present study is the first to adopt a network approach to focus on the correlations among the Pain Catastrophizing Scale (PCS), Fear of Progression (FoP), and Tampa Scale for Kinesiophobia (TSK) in AMI patients. The network analysis results showed that the item “Exercise aggravates heart problems (TSK2)” had the highest expected influence (EI = 1.279) and was the core symptom within the entire network. This indicates that TSK2 plays a pivotal role in changing the states of other nodes in the network. This symptom is reflected in patients' belief that participating in exercise or physical work will deteriorate their heart conditions. This finding is consistent with previous research on the fear responses of cardiac patients (28). It should be emphasized that high expected influence in network analysis reflects strong connectivity and predictive power in the cross-sectional network, not causal importance. Thus, the identified core symptom should be regarded as a key screening clue rather than a direct intervention target. Causal evidence from longitudinal or intervention simulation studies is needed before confirming clinical intervention priorities.

Studies have shown that age typically has an impact on the quantity and quality of health education that patients receive (29). As patients age, they are more likely to develop a stronger fear of exercise and become increasingly reluctant to engage in it. Moreover, almost all patients who have an aversion to exercise experience varying degrees of anxiety, which may be related to the duration of their hospital stay and insufficient access to health education. Additionally, some patients do not understand the importance of rehabilitation exercise, hold negative attitudes and beliefs about it, and lack adequate social support (30). As a result, they show a certain degree of avoidance of postoperative rehabilitation exercise and are resistant to carrying it out. Focusing on the activation of this specific symptom may potentially lead to the activation of other symptoms in the network. Once these symptoms are activated, they can trigger a cascade effect, affecting more symptoms and spreading throughout the entire network.

Healthcare professionals ought to closely monitor this symptom. Drawing on cognitive-behavioral therapy (31), they can implement staged cognitive and behavioral interventions for patients who have undergone percutaneous coronary intervention (PCI) postoperatively. This intervention process should incorporate the use of existing social media technology, enabling remote communication with discharged patients, and a prompt evaluation of the intervention's efficacy should be conducted. Meanwhile, it is crucial to understand patients' needs. Depending on the individual circumstances of each patient, suitable online and offline rehabilitation approaches should be offered to address issues such as the patients' transportation burden and barriers posed by weather conditions (32). Since good social support can remarkably enhance patients' motivation to exercise (33), healthcare providers should encourage patients' relatives, friends, and caregivers to show more concern for and provide greater support to the patients. They can also promote communication among patients to strengthen their confidence in recovering from the disease. Considering that 54.3% of our participants were elderly and a considerable proportion had low education levels, simplified, visualized, and repeated health education should be provided. In addition, family members should be involved in rehabilitation guidance to improve understanding and compliance. Low-intensity and safe exercises such as slow walking, seated limb activities, and Tai Chi are recommended to gradually build patients' confidence and reduce the misconception that exercise will aggravate heart problems.

The results demonstrated that the strongest association existed between “When I am anxious, I will experience some physical discomforts, such as a rapid heartbeat, stomachache, and nervousness (FoP5)” and “I may have to rely on strangers for my daily life, which makes me anxious (FoP7)”, which is consistent with previous studies (34). Anxiety during hospitalization influences the level of patients' fear of disease progression, and the two are positively correlated. Simultaneously, the fear of disease progression exacerbates negative emotions, giving rise to heightened feelings of loneliness and helplessness. This can lead to a negative state of avoidance, where patients refuse to participate in social activities, ultimately resulting in social alienation (35). Health anxiety has a detrimental impact on disease acceptance among AMI patients, giving rise to negative attitudes and emotions, which in turn affects the recovery outcomes. Patients often worry that their disease will deteriorate further and lead to more severe consequences. This fear increases patients' stress and anxiety levels, thereby further impeding their recovery process (36). Patients with health anxiety often struggle to accept the reality of their illness and may resist treatment and rehabilitation measures. This anxiety affects the patients' psychological state and rehabilitation outcomes, which subsequently influences their acceptance of the disease (37). This implies that patients with health anxiety are more prone to experiencing fear and anxiety regarding the progression of the disease. Moreover, the patients' perception of the disease also plays a role in influencing their fear of disease progression (38). A positive perception of the disease can improve the patients' psychological state and correct their irrational behavioral patterns and cognitive biases. Conversely, a poor perception of the disease reduces the patients' self-perception of health, increases adverse emotional reactions triggered by the disease, and exacerbates feelings of anxiety as well as negative and incorrect mental states, thereby intensifying the patients' fear.

It has been shown (39) that the fear of disease progression can be directly reduced by alleviating the patient's symptom burden. After falling ill, patients experience a variety of physical, cognitive, psychological, and other symptoms that trigger the development of fear. Therefore, healthcare professionals should develop tailored health education materials for AMI patients with significant symptoms. These resources can assist patients in better understanding their condition, restoring confidence in their recovery, and reducing anxiety regarding disease progression. Healthcare providers can educate patients about the psychology behind disease-related anxiety. By providing health education, they can clarify the relationship between the disease and the patients' psychological state, encourage patients to vent their negative emotions in a reasonable manner, and decrease the level of fear of disease progression (40). Personalized psychological support is crucial at different stages of a patient's illness (41). Healthcare professionals can guide patients to explore how their own thoughts influence their behaviors and emotions. By teaching patients to recognize that “the problem is not the problem itself, but rather how they respond to it”, professionals can help patients regain psychological flexibility and promote positive coping strategies. Future research is warranted to further examine bridging symptoms identified in the present network. These bridging symptoms may serve as potential early warning indicators rather than direct intervention targets, given that statistical centrality does not imply causal priority. Prospective or intervention simulation studies are needed to verify whether timely identification of highly connected bridging symptoms, together with appropriate psychological support, could help reduce symptom activation and interrupt negative symptom cascades. Such efforts may improve the efficiency and precision of clinical assessment and supportive care.

The results showed that the strongest associations between the Fear of Progression (FoP) and Pain Catastrophizing Scale (PCS) were observed for “I am afraid of the pain caused by this disease” (FoP3) and “I keep thinking back to another painful experience” (PCS7), which is consistent with previous studies (42). AMI patients, threatened by pain, often have recurrent thoughts about cardiac problems, which generate false perceptions and subsequently trigger adverse emotions such as anxiety. Especially when the pain recurs (43), patients struggle to tolerate the organic damage caused by severe pain and strongly desire the intense pain to subside quickly to relieve their discomfort. Studies have found (44) that pain catastrophizing has a certain incidence among coronary artery disease patients and is closely related to the degree of pain experienced. Additionally, the degree of pain, cardiac function grading, and gender also influence patients' pain perception. Specifically, the longer the disease duration (45) and the higher the pain intensity, the higher the patients' pain catastrophizing scores. A qualitative study of cardiac patients (46) revealed that recalling a heart attack increased patients' fear and distress. This may be because trauma—centered rumination heightens their fear and sadness (47) while reinforcing their negative beliefs about the trauma, thereby exacerbating patients' fear of illness.

Healthcare professionals can manage this symptom by intervening in neighboring symptoms within the network. In clinical practice, these relevant symptoms should be identified, and the patient's pain should be closely monitored. Based on addressing the source of the pain stimulus, a comprehensive treatment program encompassing emotional regulation, cognitive adjustment, and exercise therapy, such as acceptance and commitment therapy (48), should be implemented. Additionally, integrating aerobic exercises like Baduanjin and Taijiquan (49) can help alleviate the patient's disease burden and mitigate the impact of pain. Some studies (50) have proposed that a team—managed care model can enhance health education through collaborative efforts among healthcare team members. This approach improves patients' understanding of the disease and nutrition, enhances their nutritional status, reduces disease—related fear, and boosts their confidence in combating the illness. Therefore, healthcare professionals can establish a scientific and efficient team—based nursing model. Appropriate health education should be provided to patients during hospitalization and before discharge. Regular telephone follow—ups after discharge can be conducted to communicate with patients, thereby establishing an effective follow—up health education mechanism.

The strengths of this study are multi—faceted. Firstly, the application of network analysis represents a significant methodological advancement. By visualizing the fear network of acute myocardial infarction patients, this approach offers a novel and intuitive way to map out the complex interconnections among various fear—related symptoms. It successfully identifies the core symptoms of patients' fear, which is crucial for understanding the underlying mechanisms of patients' psychological distress. This not only enables healthcare professionals to grasp the specific roles and interactions between different fears but also provides new perspectives for exploring the intricate relationships among these fears. As a result, it serves as a valuable guide for the development and implementation of clinical precision intervention programs, potentially leading to more targeted and effective patient care.

However, the study is not without limitations. First, although the CS-coefficient demonstrated acceptable stability, a sample size of 449 may still be relatively limited for a high-dimensional network with over 40 nodes, which may reduce the replicability of weaker edges and requires cautious interpretation. One of the major drawbacks is its cross—sectional design. This type of study, by nature, can only capture the situation at a single point in time, thereby precluding the exploration of causal relationships between specific fear symptoms. Without the ability to establish cause—and—effect links, it becomes challenging to fully understand the development and progression of these fears, which may limit the long—term impact of the findings on clinical practice. Although inter-item correlations were low in the current study, partial conceptual overlap between items derived from different scales cannot be fully excluded. Future research may therefore consider exploratory factor analysis or dimensionality reduction methods to refine the item pool for network modeling. Additionally, the study was conducted in only one tertiary hospital. This single—center nature of the research restricts the sample size and diversity. The sample may not be representative of the entire population of acute myocardial infarction patients, as patients from different geographical locations, socioeconomic backgrounds, and healthcare systems may have different experiences and manifestations of fear. This limitation reduces the generalizability of the study results and may lead to biased conclusions.

## 5 Conclusion

This network analysis identified core and bridging fear symptoms among post-PCI AMI patients. The symptom “Exercise aggravates heart problems” showed the highest expected influence and may serve as an exploratory indicator for clinical screening; clinical applications require future longitudinal or intervention studies. However, statistical centrality does not represent causality, so these findings should be considered as clues for assessment rather than definite intervention targets. Tailored interventions based on patients' age and literacy levels, combined with low-intensity exercises, may help reduce fear and promote cardiac rehabilitation.

## Data Availability

The original contributions presented in the study are included in the article/Supplementary Material, further inquiries can be directed to the corresponding author.

## References

[1] Summary of the China cardiovascular health and disease report 2023. Chin J Circ. (2024) 39(7):625–60.

[2] Hoole SP Bambrough P. Recent advances in percutaneous coronary intervention. Heart. (2020) 106(18):1380–6. 10.1136/heartjnl-2019-315707 32522821

[3] Ma WJ Ma HP Wang YH Wang J Wang HJ Wang SZ. Summary of the report on the quality of cardiovascular care in China in 2021. Chin J Circ. (2021) 36(11):1041–64.

[4] Correction to: 2024 heart disease and stroke statistics: a report of US and global data from the American Heart Association. Circulation. (2023) 149(19):e1164. 10.1161/CIR.0000000000001247 38709844

[5] Xu QF Chen J Zhao JM Wang ZL. Regulation of CRP, BNP and cardiac function indexes in patients after percutaneous coronary intervention for acute myocardial infarction with conventional therapy combined with early cardiac rehabilitation. J Med Forum. (2022) 43(4):70–3.

[6] Henry TD Tomey MI Tamis-Holland JE Thiele H Rao SV Menon V. Invasive management of acute myocardial infarction complicated by cardiogenic shock: a scientific statement from the American Heart Association. Circulation. (2021) 143(15):e815–29. 10.1161/CIR.0000000000000959 33657830

[7] Chen Q Zang S. Effects of applying motivational psychological intervention under the focused solution model on compliance behavior and prognosis in patients with acute myocardial infarction in the emergency intensive care unit. Chin J Health Psychol. (2021) 29(6):866–72.

[8] Su J Yang QH Li YX Xiong JM Qiu WY. Analysis on classification and influencing factors of fear of disease progression in young and middle-aged patients with acute myocardial infarction during early rehabilitation. Chin J Nurs. (2023) 58(4):406–13.

[9] Qin J Xiong J Chen C Wang X Gao Y Zhou Y. Influencing factors of kinesiophobia in older patients with chronic heart failure: a structural equation model. Clin Cardiol. (2023) 46(7):729–36. 10.1002/clc.24024 37114367 PMC10352966

[10] Ravn SL Vang ML Vaegter HB Andersen TE. Pain-related acceptance as a mediator in the fear avoidance model of chronic pain: a preliminary study. Pain Med. (2018) 19(9):1764–71. 10.1093/pm/pnx223 29036699

[11] Vlaeyen JWS Linton SJ. Fear-avoidance and its consequences in chronic musculoskeletal pain: a state of the art. Pain. (2000) 85(3):317–32. 10.1016/S0304-3959(99)00242-0 10781906

[12] Wu DQ Zhang Y Qian QY Gu Y Zhao HP Xu BB. Relationship between fear of disease progression and rehabilitation level in patients with acute myocardial infarction: a mediating effect analysis based on coping styles. Gen Pract Nurs. (2024) 22(9):1755–9.

[13] Kori S. Kinesiophobia: a new view of chronic pain behaviour. Pain Manag. (1990) 3:35–43.

[14] Dankert A Duran G Engst-Hastreiter U Keller U Waadt M Henrich S. Fear of progression in patients with cancer, diabetes mellitus and chronic arthritis. Rehabilitation. (2003) 42(3):155–63. 10.1055/s-2003-40094 12813652

[15] Petrini L Arendt-Nielsen L. Understanding pain catastrophizing: putting pieces together. Front Psychol. (2020) 11:603420. 10.3389/fpsyg.2020.603420 33391121 PMC7772183

[16] Wang YX Sang WF Jia GH Qin NN Sun JJ. Analysis on the status quo and influencing factors of kinesiophobia in patients with first-ever acute myocardial infarction. Chin Nurs Manag. (2022) 22(1):63–9.

[17] Briganti G Scutari M Epskamp S Borsboom D Hoekstra RHA Golino HF. Network analysis: an overview for mental health research. Int J Meth Psych Res. (2023) 33(4):e2034. 10.1002/mpr.2034 PMC11564129 39543824

[18] Hofmann SG Curtiss J McNally RJ. A complex network perspective on clinical science. Perspect Psychol Sci. (2016) 11(5):597–605. 10.1177/1745691616639283 27694457 PMC5119747

[19] Borsboom D Cramer AO. Network analysis: an integrative approach to the structure of psychopathology. Annu Rev Clin Psycho. (2013) 9:91–121. 10.1146/annurev-clinpsy-050212-185608 23537483

[20] Yap JC Lau J Chen PP Gin T Wong T Chan I. Validation of the Chinese pain catastrophizing scale (HK-PCS) in patients with chronic pain. Pain Med. (2008) 9(2):186–95. 10.1111/j.1526-4637.2007.00307.x 18298701

[21] Bäck M Cider Å Herlitz J Lundberg M Jansson B. The impact on kinesiophobia (fear of movement) by clinical variables for patients with coronary artery disease. Int J Cardiol. (2013) 167(2):391–7. 10.1016/j.ijcard.2011.12.107 22305808

[22] Mengjie L Tingting L Siqi X Henrich G Koch U. Sinicization and reliability validity test of exercise fear scale for cardiac patients. Chin Nurs Manag. (2019) 19(11):1637–42.

[23] Mehnert A Herschbach P Berg P Henrich G Koch U. Fear of progression in breast cancer patients–validation of the short form of the fear of progression questionnaire (FoP-Q-SF). Z Psychosom Med Psyc. (2006) 52(3):274–88. 10.13109/zptm.2006.52.3.274 17156600

[24] Wu QY Ye ZX Li L. Sinicization and reliability analysis of simplified fear of progression questionnaire-short form for cancer patients. Chin J Nurs. (2015) 50(12):1515–9.

[25] Zhu Z Sun Y Kuang Y Yuan X Gu H Zhu J. Contemporaneous symptom networks of multidimensional symptom experiences in cancer survivors: a network analysis. Cancer Med. (2023) 12(1):663–73. 10.1002/cam4.4904 35651298 PMC9844664

[26] Bringmann LF Elmer T Epskamp S Krause RW Schoch D Wichers M. What do centrality measures measure in psychological networks? J Abnorm Psychol. (2019) 128(8):892–903. 10.1037/abn0000446 31318245

[27] Borsboom D Deserno M Rhemtulla M Epskamp S Fried EI McNally RJ. Network analysis of multivariate data in psychological science. Nat Rev Methods Primers. (2021) 1(1). 10.1038/s43586-021-00055-w

[28] Zhou Y Gao X Xu J Ding X Yuan J Du S. Network analysis of perception of exercise benefits/barriers and kinesiophobia among patients with cardiovascular diseases. Heart Lung. (2024) 64:182–8. 10.1016/j.hrtlng.2023.12.006 38281371

[29] Wang Z Zhang Y Wang Y Liu L Zhang J. Kinesiophobia and its associated factors in patients with coronary heart disease: a cross-sectional study based on latent feature analysis. BMJ Open. (2023) 13(7):e072170. 10.1136/bmjopen-2023-072170 37429691 PMC10335492

[30] Keessen P Latour CHM van Duijvenbode ICD Visser B Proosdij A Reen D. Factors related to fear of movement after acute cardiac hospitalization. BMC Cardiovasc Disord. (2020) 20(1):495. 10.1186/s12872-020-01783-9 33228521 PMC7686769

[31] Cai L Gao H Xu H Wang Y Lyu P Liu Y. Does a program based on cognitive behavioral therapy affect kinesiophobia in patients following total knee arthroplasty? A randomized, controlled trial with a 6-month follow-up. J Arthroplasty. (2018) 33(3):704–10. 10.1016/j.arth.2017.10.035 29239772

[32] Su YH Huang Y Liu LL Liang JB. The mediating effect of kinesiophobia between health literacy and cardiac rehabilitation barriers in patients with coronary artery disease after PCI. J Pract Cardiovasc Pulm Vasc Dis. (2025) 33(2):124–9.

[33] Li HS Wu J Dong FW Zhang J Huang L Li XY. Latent profile analysis of kinesiophobia in patients with atrial fibrillation after radiofrequency ablation. J Nurs. (2023) 38(10):43–46+64.

[34] Deng XM Hou LY Shi X Peng WQ Wu M Deng XL. Correlation analysis of fear of disease progression with anxiety and depression among hospitalized patients. Gen Pract Nurs. (2021) 19(34):4866–9.

[35] Shi D Li R Chen P Zhong JQ Wang KL Wang D. The mediating effect of stigma on the relationship between fear of disease progression and social alienation in patients with haematological malignancies. Hematology. (2024) 29(1):2416723. 10.1080/16078454.2024.2416723 39466116

[36] Wang Y Wu Z. The effect of fear of disease progression on the readiness of young and middle-aged chronic heart failure patients to return to work. Cardiovasc Dis Prev Control. (2022) 22(3):80–2.

[37] Xu B Ber X. Correlation analysis of health anxiety metacognitive level with disease acceptance and fear of disease progression in hemiplegic patients after stroke. J Pract Cardiovasc Pulm Vasc Dis. (2024) 32(11):131–6.

[38] Liu QW Qin T Hu B Zhao YL Zhu XL. Relationship between illness perception, fear of progression and quality of life in interstitial lung disease patients: a cross-sectional study. J Clin Nurs. (2021) 30(23–24):3493–505. 10.1111/jocn.15852 33998090

[39] Liu R Liu J Song J Peng Y Jin G Li J. Mediating effect of social support in the relationship of symptom burden and fear of disease progression in stroke patients. J Stroke Cerebrovasc. (2025) 34(3):108215. 10.1016/j.jstrokecerebrovasdis.2024.108215 39743003

[40] Zhu J Han X. Analysis of fear of disease progression status in patients with acute myocardial infarction after emergency PCI. J Clin Psychosom Dis. (2022) 28(6):112–5.

[41] Wang X Liu M Li J Wang Z Liang Q Yan Z. Relationship between quality of life, fear of disease progression, and coping styles in patients with pulmonary hypertension: a network analysis. Res Nurs Health. (2023) 46(5):546–57. 10.1002/nur.22333 37537879

[42] Wang YY Zhang J Sun YJ Bai YJ Xu HF. Chain mediating effects of pain catastrophizing and psychogenic anxiety between pain and kinesiophobia in patients with acute coronary syndrome. J Nurs Manag. (2024) 24(2):103–7.

[43] Häggman-Henrikson B Visscher CM Wänman A Ljótsson B Peck CC Lövgren A. Even mild catastrophic thinking is related to pain intensity in individuals with painful temporomandibular disorders. J Oral Rehabil. (2021) 48(11):1193–200. 10.1111/joor.13251 34462940

[44] Wang RH Wang Y Li Y Fu X. Current status of pain catastrophizing and its influencing factors in patients with coronary atherosclerotic heart disease. PLA Nurs J. (2022) 39(2):21–4.

[45] Chen Y Ju P Xia Q Cheng P Gao J Zhang L. Potential role of pain catastrophic thinking in comorbidity patients of depression and chronic pain. Front Psychiatry. (2022) 13:839173. 10.3389/fpsyt.2022.839173 35898637 PMC9309267

[46] Liang Z Lin S Sun H Liao Y Zhang M Chen C. A qualitative study exploring the experiences and needs of fear of disease progression in patients after open-heart surgery. J Psychosom Res. (2025) 188:111980. 10.1016/j.jpsychores.2024.111980 39549654

[47] Wisco BE Vrshek-Schallhorn S May CL Campbell AA Nomamiukor FO Pugach CP. Effects of trauma-focused rumination among trauma-exposed individuals with and without posttraumatic stress disorder: an experiment. J Trauma Stress. (2023) 36(2):285–98. 10.1002/jts.22905 36655347

[48] Zhuang X Zhu C. A study on the application of acceptance and commitment therapy in patients with lumbar disc herniation. J Nurs Manag. (2021) 21(11):825–8.

[49] Ding LP Fan XR Liu F Wei G Ren J Xu F. Network meta-analysis of different aerobic exercise modalities for improving cardiac function in patients with coronary heart disease. J Nurs Manag. (2023) 23(2):77–82.

[50] Li L Liu Y Li X He Y Tian Z. Effect of group management on disease cognition and fear of disease progression, nutritional status, and quality of life in patients with head and neck tumors. Am J Transl Res. (2024) 16(12):7937–47. 10.62347/CBKG3767 39822501 PMC11733339

