# Supplementary material for: Network analysis identifies core fear symptoms as screening clues for post-PCI rehabilitation in patients with acute myocardial infarction
Source: Front Cardiovasc Med. 2026 Jun 15;13:1805006. doi: 10.3389/fcvm.2026.1805006 (PMC13310681; doi:10.3389/fcvm.2026.1805006)
Supplement: Supplementary file 1 [file Datasheet1.docx]

***Supplementary Material***

***
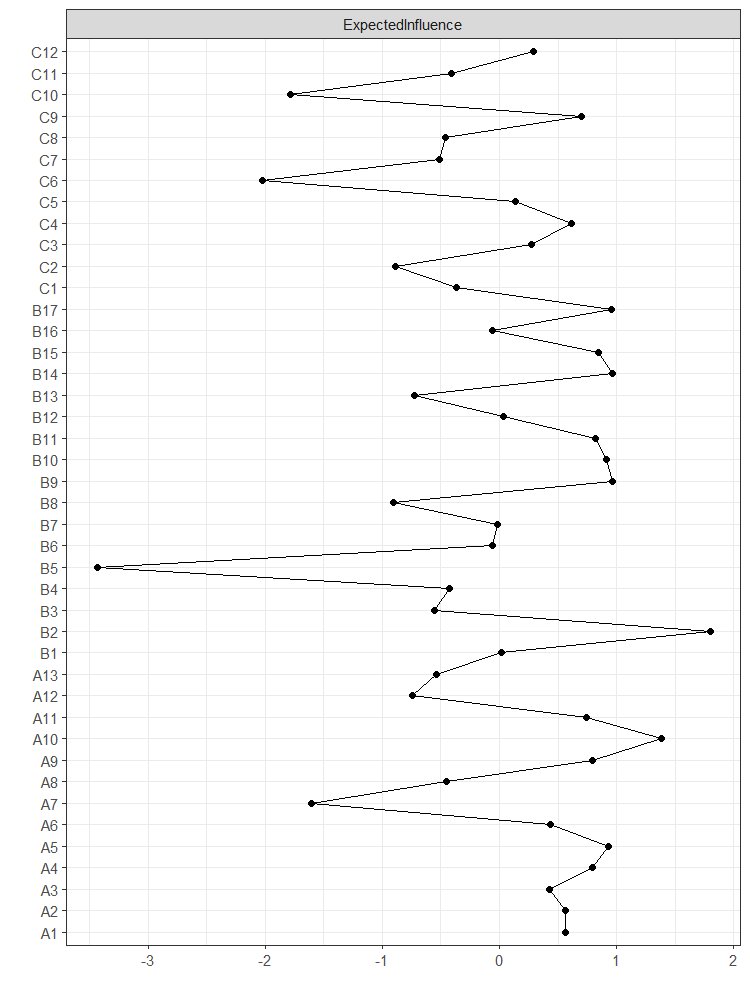
***

**Supplementary Figure 1. Node expected influence in the network (z-score).**

Node expected influence represents the centrality of the nodes. A higher centrality indicates the node has stronger connections to other nodes.

***
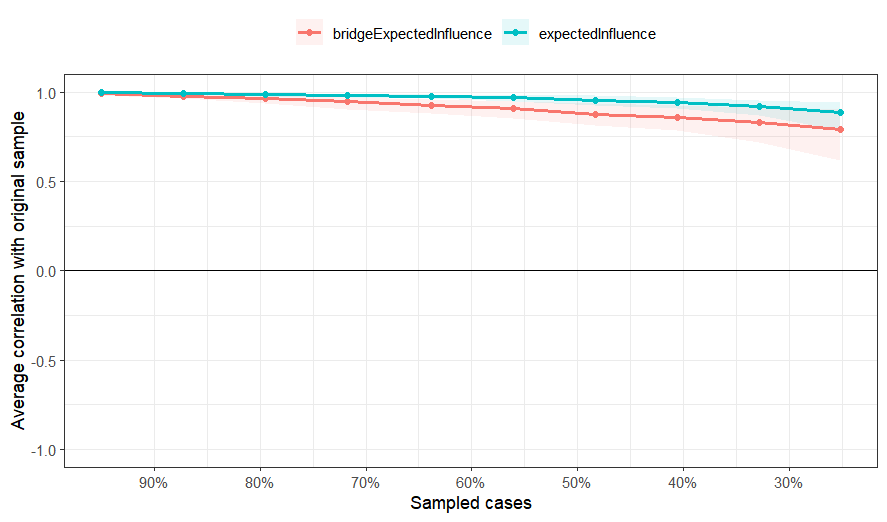
***

**Supplementary Figure 2. Stability of centrality indices by case dropping subset bootstrap.**

The x-axis represents the percentage of cases in the original sample used at each step. The y-axis represents the average of correlations between the centrality indices in the original network and the centrality indices in the networks that were re-estimated after dropping increasing percentages of cases. Color areas indicate 95% CI.

***
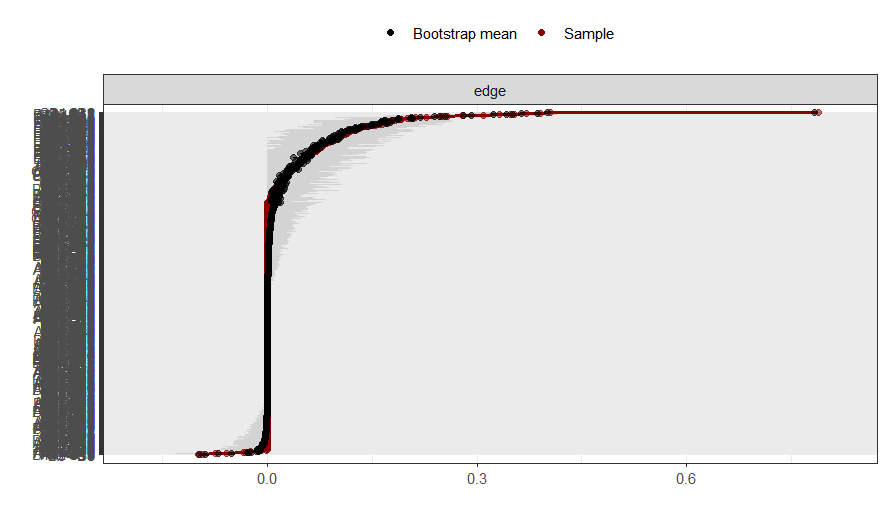
***

**Supplementary Figure 3. Bootstrapped 95%CIs of estimated edge weights**

Note: The red dots indicate the values of each edge weight, ordered from the highest to the lowest edge-weight values. The gray area represents the 95% CIs of edge weights, estimated with the non-parametric bootstrap procedure. Wide intervals indicate lower stability and narrow intervals indicate higher stability.

***
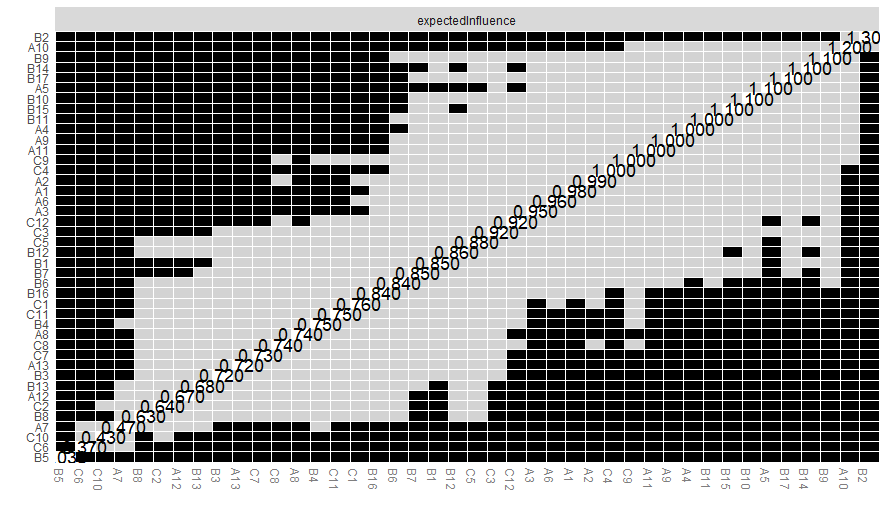
***

**Supplementary Figure 4. Nonparametric bootstrapped difference test for expected influence**

Note: Gray boxes indicate no difference between nodes, whereas black boxes indicate significant difference (α = 0.05). Values reported in the diagonal represent the expected influence values of each node.
